# A method for feature division of Soccer Foul actions based on salience image semantics

**DOI:** 10.1371/journal.pone.0322889

**Published:** 2025-06-13

**Authors:** Jianming Wang, Lifeng Li

**Affiliations:** 1 Department of Physical Education, Tongling University, Tongling, Anhui, China; 2 Sports Training Institute, Shenyang Sport University, Shenyang, Liaoning, China.; Government Law College, INDIA

## Abstract

The purpose of this study is to realize the automatic identification and classification of fouls in football matches and improve the overall identification accuracy. Therefore, a Deep Learning-Based Saliency Prediction Model (DLSPM) is proposed. DLSPM combines the improved DeepPlaBV 3+architecture for salient region detection, Graph Convolutional Networks (GCN) for feature extraction and Deep Neural Network (DNN) for classification. By automatically identifying the key action areas in the image, the model reduces the dependence on traditional image processing technology and manual feature extraction, and improves the accuracy and robustness of foul behavior identification. The experimental results show that DLSPM performs significantly better than the existing methods on multiple video motion recognition data sets, especially when dealing with complex scenes and dynamic changes. The research results not only provide a new perspective and method for the field of video motion recognition, but also lay a foundation for the application in intelligent monitoring and human-computer interaction.

## 1. Introduction

In modern football matches, accurate and timely foul detection is crucial to ensure fairness and fluidity in the game. With the advancement of technology, particularly the rapid development of deep learning techniques, automated foul detection and recognition systems have increasingly become a research focus [1–3]. These systems aim to assist referees in making faster and more accurate judgments to enhance the fairness and entertainment value of the game. This study introduces a novel deep learning-based saliency prediction model (DLSPM), which automatically identifies salient regions in images, combines them with graph convolutional networks (GCN) to extract features within these regions, and then utilizes deep neural networks to identify and classify different types of foul actions. The development of this method aims to address many limitations of traditional approaches to foul action recognition in football matches and provide robust technical support for referees.

Traditional methods for identifying and classifying foul actions in football matches typically rely on simple image processing techniques and manual feature extraction, such as edge detection, optical flow estimation, and template matching [4–6]. While these methods have achieved some degree of success in early research and applications, they face significant challenges when dealing with complex scenarios. Particularly when encountering issues like occlusion, rapid motion, and background interference, the effectiveness of these methods diminishes considerably. Moreover, traditional methods are quite sensitive to image noise, changes in lighting conditions, and scene diversity, leading to a significant decrease in their accuracy and robustness in practical applications. In addition to traditional image processing methods, deep learning techniques are beginning to be widely used in the field. For example, some recent studies have utilized convolutional neural network (CNN) and recurrent neural network (RNN) to improve the recognition accuracy of foul play. In addition, Transformer-based approaches have also emerged in the field of action recognition. Models such as Visual Transformer (ViT) and DEtection TRansformer (DETR) can efficiently capture long-distance dependencies through the self-attention mechanism, thus performing well in complex scenes. However, these approaches still face challenges in dealing with complex scenes and dynamic relationships.

To address these challenges, the motivation of this study lies in leveraging the powerful capabilities of deep learning to overcome the limitations of traditional methods. Deep learning has demonstrated remarkable performance in the fields of image recognition, processing, and semantic analysis, as it can automatically learn useful feature representations from complex data. Therefore, the proposed DLSPM method aims to integrate the latest advances in saliency prediction and image semantic understanding using deep learning models to automatically identify key actions in images and accurately classify foul types. At the core of this method is the utilization of deep learning models to first identify salient regions in images and then employ graph convolutional networks to analyze the features and structures within these regions comprehensively. Finally, through deep neural networks, the method achieves precise recognition and classification of foul actions. By combining saliency prediction and semantic understanding within a deep learning framework, this approach enhances the ability to detect and classify foul actions in football matches, surpassing the limitations of traditional methods.

The contributions of this study are highlighted as follows

◽ We introduce an innovative method for the detection and classification of foul play in soccer matches, employing a Deep Learning-based Salience Prediction Model (DLSPM) that autonomously identifies salient regions within images.

◽ Our approach leverages Graph Convolutional Networks (GCN) for an in-depth feature analysis within these salient regions, enhancing the accuracy and robustness of foul play classification.

◽ The methodology significantly reduces reliance on manual feature extraction, improving the system’s automation and efficiency by learning complex image characteristics and semantic information directly from the data.

◽ Experimental results on diverse football match datasets demonstrate superior performance in terms of accuracy and robustness compared to existing methods, underscoring the practical utility of our approach in supporting fair and precise officiating in soccer.

## 2. Related works

In the field of automated foul detection in football, numerous scholars have proposed various methods and approaches, aiming to improve the accuracy and robustness of foul recognition. Below is a more detailed review of the relevant works:

The endeavors delineated in [6–10] primarily hinge on traditional image processing techniques as the bedrock for identifying foul actions during football matches. These methodologies lean on methodologies such as edge detection, optical flow estimation, and motion trajectory analysis, endeavoring to discern foul situations through the meticulous scrutiny of players’ dynamic movements. Despite their commendable performance in straightforward scenarios, these approaches falter when confronted with the complexities inherent in intricate backgrounds, swift player maneuvers, and obstructive occlusions. Moreover, they often grapple with the task of effectively delineating nuanced differences in motion, thereby curtailing their utility in the nuanced adjudication of intricate foul scenarios.

Advancements in deep learning, as delineated in [11–15], have heralded the exploration of deep convolutional neural networks (CNNs), recurrent neural networks (RNNs), long short-term memory networks (LSTMs), among other architectures, with the aim of fortifying the efficacy of foul action recognition. These studies, through the assimilation of copious match video data, have markedly enhanced the accuracy and resilience of foul recognition. Noteworthy is the concerted effort in some studies to amalgamate the strengths of diverse models—such as the spatial feature extraction process of CNNs coupled with the temporal motion analysis capabilities of RNNs or LSTMs—in a bid to attain superior recognition outcomes. Nonetheless, these deep learning methodologies often necessitate substantial annotated datasets and are encumbered by their limited interpretability, thereby necessitating further refinement to enhance their adaptability to real-world application scenarios.

To circumvent the challenges posed by background clutter and the complexities of recognition in multifaceted scenes, the integration of saliency detection techniques, as expounded in [16–20], has been pivotal. By singling out pivotal regions within images or video frames, these approaches mitigate the interference of extraneous background elements, thereby refining the accuracy of foul action recognition. Moreover, select studies [21–23] have embarked on leveraging GCN to scrutinize player interactions and the dynamic interplay within the scene, thus furnishing a richer contextual understanding for action recognition. By establishing a relational graph among players, GCNs can unearth deeper insights into action features and interaction patterns, thereby presenting a novel paradigm for foul action classification.

In addition to the above studies, GCNs have been increasingly used in image processing and computer vision. For example, the Face2Nodes method studied by Jiang et al. (2023) [24] utilized relationally aware dynamic graph convolutional networks to learn facial expression representations with remarkable results. In addition, the bi-graph attentional convolutional network for 3D point cloud classification studied by Huang et al. (2022) [25] demonstrated the power of GCNs in processing complex data. These studies provide strong support for the application of GCN in the recognition of foul behavior in soccer games.

In recent scholarship, deeper forays into the amalgamation of deep learning methodologies with image semantic analysis techniques have been witnessed. These approaches, beyond merely attending to players’ motion dynamics, endeavor to unravel the semantic intricacies inherent in scenes by parsing objects, action interrelations, and their semantic connotations. Such endeavors aspire to furnish more nuanced and precise judgments regarding foul situations. Nevertheless, the efficient amalgamation of these methodologies, coupled with the resolution of compatibility and information fusion challenges across disparate models, remains an ongoing endeavor within contemporary research pursuits.

In summary, while there have been advancements in the field of automated foul detection in football, effectively handling rapid motion changes, occlusion, background interference in complex scenes, and accurately understanding semantic information within the scene remain major challenges. In comparison to the methods mentioned earlier, the DLSPM proposed in this paper exhibits distinct differences and advantages:

Firstly, DLSPM reduces reliance on traditional image processing techniques and manual feature extraction by automatically identifying salient regions in images through deep learning. This automated saliency detection not only enhances the efficiency of the recognition process but also improves the model’s adaptability and robustness to complex scenes. Compared to traditional methods relying on optical flow estimation or motion trajectory analysis, DLSPM can more accurately identify and focus on key actions and regions in the match. Secondly, DLSPM incorporates GCN to analyze features within salient regions and interactions between players, a feature less common in previous research. The introduction of GCN enables DLSPM to capture dynamic relationships between players and deeper semantic information within the scene, thereby achieving higher accuracy in foul action recognition and classification. Lastly, DLSPM integrates deep neural networks to identify and classify different types of foul actions, distinguishing it from existing methods that rely solely on single models or techniques. By leveraging the advantages of saliency detection, image semantic analysis, and deep learning, DLSPM provides a comprehensive solution to address the complexity and diversity of foul recognition in football matches.

## 3. Deep learning-based saliency prediction model

**Fig 1:**
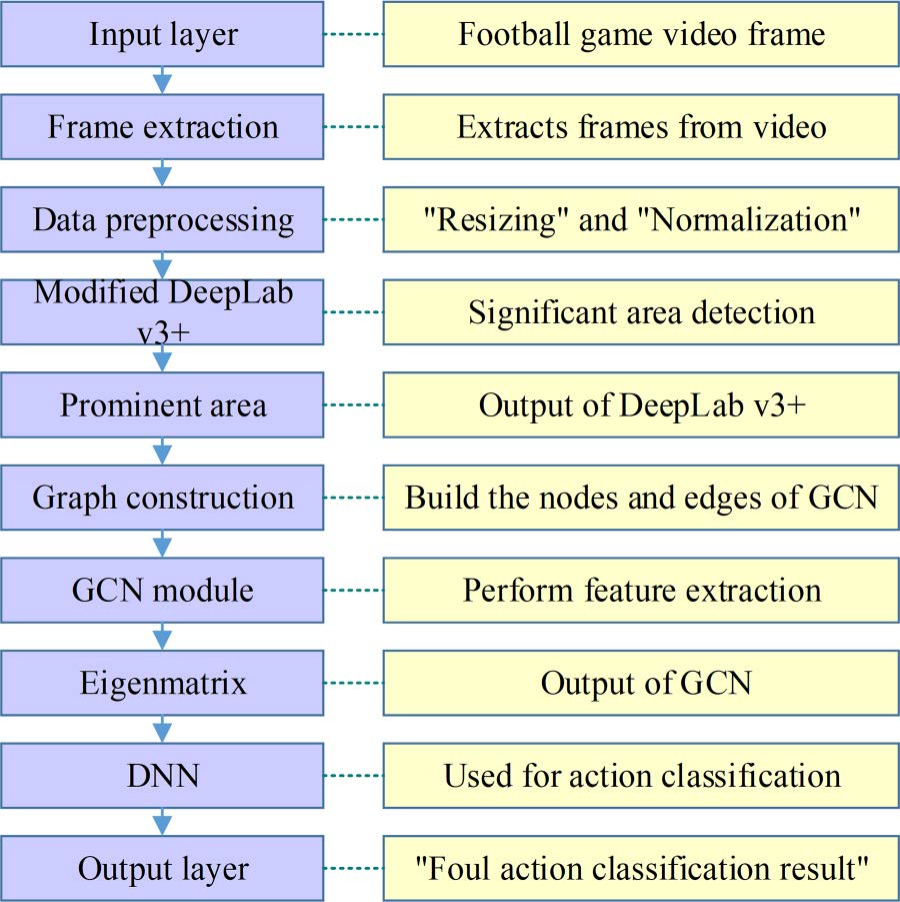
Overall framework of DLSPM.

In this section, the methodology behind the DLSPM aimed at distinguishing features of foul play actions in soccer games is introduced. The approach encompasses three main phases: data preparation and preprocessing, saliency region detection, and action classification. Initially, soccer match videos are collected and processed to extract relevant frames, which are then annotated for specific types of fouls. These preprocessed frames are fed into a saliency detection model to identify regions of interest, which are subsequently analyzed using GCN to extract features that are essential for classifying the type of foul action. The final classification is performed by a deep neural network trained on these features. Fig 1 summarizes the overall framework of DLSPM proposed in this study.

### 3.1. Data preparation and preprocessing

In this section, firstly, the process of data collection and preprocessing is introduced, including the game videos obtained from multiple leagues, and the video frames are marked and preprocessed. All the video data are from the open database of sports events, and have obtained the corresponding authorization or permission. This study ensures that all the video data used are legally obtained and do not infringe any copyright or privacy rights. When collecting and using video data, strict privacy protection policies are followed. All involved athletes and related personnel have obtained clear informed consent to use their competition videos for scientific research purposes. This study has been approved by the ethical review of Moral Committee of Tongling University, with the approval number of No.20221116B. All research activities follow the guiding principles of the Ethics Review Committee and relevant laws and regulations.

*Data collection*: The data comprises video recordings from various soccer leagues and seasons, amounting to a total of 200 matches with over 500 instances of foul play. These videos were sourced from public sports event databases and collaborations with soccer clubs, ensuring a diverse and comprehensive dataset. Frames were extracted from the videos at a rate of 10 frames per second using automated scripts, capturing the continuity and details of the actions. The game videos in the data set come from several top football leagues, including English Premier League (EPL), La Liga, Bundesliga, Serie A and Ligue 1. These leagues represent the highest level of football matches in the world, ensuring the universality and representativeness of the data. In addition to common fouls, the dataset also contains some rare types of fouls, such as diving and delaying the game time. The addition of these rare behaviors further enriches the diversity of data sets and helps the model to better identify foul behaviors in complex situations. LabelMe is used as the main marking tool, which allows the marking personnel to mark the areas related to the foul behavior in the video frame and specify the specific foul type (such as handball, tackle, etc.) for each area. LabelMe also supports polygon labeling, which can accurately capture areas with irregular shapes.

### Data annotation

The annotation of foul play actions was carried out by professional soccer referees and sports analysts, ensuring accuracy and consistency in the dataset. Annotations included the type of foul (e.g., handball, sliding tackle) and the specific timeframe of the action within the video. Each foul event was pinpointed to exact frames, facilitating subsequent saliency detection and feature extraction.

### Preprocessing steps:

The extracted frames underwent several preprocessing steps to standardize the input for the model. This included resizing the images to a uniform resolution of 256x256 pixels and normalizing the pixel values to fall within the range of 0–1. The normalization process can be represented mathematically as:


$$p^{\prime }=\frac{p-\min\left(p\right)}{\max\left(p\right)-\min\left(p\right)}$$ (1)

where p is the original pixel value, and $p^{\prime }$ is the normalized pixel value. This preprocessing reduces computational complexity and enhances the model’s processing speed.

### Color space conversion:

Frames were converted from RGB to the YUV color space, separating luminance from chrominance, to reduce the model’s sensitivity to color variations. The conversion is defined as:


Y=0.299R+0.587G+0.114B (2)

U=−0.147R−0.289G+0.436B (3)

V=0.615R−0.515G−0.100B (4)

In the data preprocessing stage, YUV color space is selected instead of RGB color space, which is based on the unique advantages of YUV color space in image processing. YUV color space divides the image into two components: brightness (Y) and chroma (U, V), in which the brightness component Y contains the main information of the image, while the chroma components U, V contain the color information of the image. This separation allows people to process and analyze the brightness components more carefully, and at the same time reduce the interference of color information on the model performance.

Specifically, choosing YUV color space is helpful to reduce the influence of illumination change on model performance. In the game scene, the lighting conditions are often complex and changeable, which will cause great interference to the color information in RGB color space. However, the brightness component Y in YUV color space is robust to illumination changes, so it can better retain the main information of the image.

### 3.2. Salient region detection

After data collection and preprocessing, the next stage aims to identify the areas that are crucial to identify and classify foul behaviors in football match images, and adopts a complex deep learning model tailored for salient feature extraction.

### Model selection

For the task of salient region detection, we employ a modified version of the DeepLabv3 + architecture, renowned for its efficiency in semantic segmentation tasks. The core of DeepLabv3 + is a CNN enhanced with atrous convolutions, allowing the model to capture multi-scale information by adjusting the field-of-view. The atrous convolution is defined as:


$$F\left(x\right)=\sum {\mathrm{\backslash nolimits}}_{k=1}^{K}w_{k}\cdot x\left[\left(r\cdot k\right)+d\right]$$ (5)

where x is the input feature map, w represents the weights of the convolutional kernel of size K, r denotes the atrous rate determining the spacing between kernel points, and d is the dilation rate.

The output of the atrous convolution feeds into an Atrous Spatial Pyramid Pooling (ASPP) module, which applies parallel atrous convolutions with varying rates to capture objects and features at different scales. The final segmentation map is generated by up-sampling the concatenated outputs of the ASPP module and combining them with features from the encoder part of the network using skip connections.

*Training process*: The model is trained on a dataset comprising annotated images from football matches, where the annotations delineate the salient regions corresponding to foul play actions. A split of 80% for training and 20% for validation is used to evaluate the model’s performance.

The loss function employed for training is a combination of the weighted cross-entropy loss for dealing with class imbalance and the Dice loss to enhance the precision of boundary detection, defined as:


$$\begin{array}{c} L=L_{1}\left(Y,\hat{Y}\right)-\log\left(Dice\left(Y,\hat{Y}\right)\right) \end{array}$$ (6)

where $L_{1}$ is the weighted cross-entropy loss, Y represents the true labels, $\hat{Y}$ denotes the predicted labels, and Dice is the Dice coefficient, a measure of overlap between the true and predicted segmentation maps.

Optimization is performed using the Adam optimizer with an initial learning rate of 1e − 4, which is adjusted according to a predetermined schedule based on the validation loss. The impact of accurate salient region detection on the task of foul play recognition is profound. By focusing on regions of interest, the subsequent classification model can operate more efficiently, leading to higher accuracy and reduced false positives in foul play detection. The salient regions detected in Section 3.2 provided the foundation for evaluating the performance metrics in Section 4. The integration of DeepLabv3+ and GCN enhanced the system’s accuracy, as discussed in detail in the following results section. The significant regions detected by the DeepLabv3 + model precisely locate the key parts related to foul actions in football match images. These regions lay the foundation for subsequent feature extraction and classification. The GCN will extract features based on these significant regions, providing key information for the DNN classification of foul actions in Section 4.1, thus improving classification accuracy and reliability.

### 3.3. Feature extraction and foul play classification

*Feature extraction*: The structure of GCN is pivotal for extracting features from the salient regions identified in football match images. Given a graph


G=(V,E) (7)

where V represents the vertices or nodes (in this case, individual pixels or segments within the salient regions) and E denotes the edges (the relationships between these segments), the GCN operates on features X associated with V to produce an output that highlights the complex, spatial relationships within data.

The feature extraction process in GCN can be formalized as:


$$H^{l+1}=\sigma \left({\tilde{D}}^{-\frac{1}{2}}\tilde{A}{\tilde{D}}^{-\frac{1}{2}}H^{l}W^{l}\right)$$ (8)

where $H^{l}$ is the feature representation at layer l, $\tilde{A}=A+I_{N}$ is the adjacency matrix A with added self-connections $I_{N}$, $\tilde{D}$ is the degree matrix of $\tilde{A}$, $W^{l}$ is the weight matrix for layer l, and σ represents a non-linear activation function such as ReLU. This formulation enables the GCN to leverage local pixel neighborhoods, effectively capturing the essence of salient features for foul play classification.

*Classification*: After feature extraction, a deep neural network (DNN) is utilized to classify the type of foul play. The network architecture is designed to accommodate the complexity and variability of the features extracted by the GCN, comprising several dense layers followed by a softmax output layer to categorize the input into predefined foul types.

The classification process can be expressed as:


$$Z=\text{softmax}\left(W_{f}H+b\right)$$ (9)

where H is the high-level feature matrix obtained from the GCN, $W_{f}$ is the weight matrix of the final dense layer, b is the bias term, and Z represents the probabilities of each foul type.

To enhance the model’s accuracy and robustness in recognizing foul plays, several optimization strategies are employed:

*Data augmentation*: By applying transformations such as rotation, scaling, and mirroring to the training images, the model’s ability to generalize from the training data to unseen images is improved.

*Regularization techniques*: L2 regularization is applied to the weights of the model to prevent overfitting, incorporated into the loss function as:


$$L_{total}=L+\lambda {\left\|W\right\|}_{2}^{2}$$ (10)

where L is the original loss, λ is the regularization coefficient, and ${\left\|W\right\|}_{2}^{2}$ represents the L2 norm of the weight matrix.

Through these methodologies, the combined use of GCN for feature extraction and DNN for classification creates a powerful framework for identifying and categorizing foul plays in football matches, leveraging the spatial and semantic complexities inherent in the task. The pseudo-code of DLSPM is shown in Table 1

**Table 1:** The pseudocode of the DLSPM.

Algorithm 1: DLSPM for Foul play detection and classification.
**Input**: Collection of Football Match Frames
**Output**: Classification of Foul Play Actions
1: **Initialize** Frame Extraction Module
2: **Initialize** Salient Region Detection Model (Modified DeepLabv3+)
3: **Initialize** GCN Model for Feature Extraction
4: **Initialize** DNN Model for Foul Play Classification
5: **for** each Match Frame in Collection do
6: Extracted Frames ← ExtractFrames(Match Frame)
7: **for** each Frame in Extracted Frames do
8: Salient Regions ← DetectSalientRegions(Frame, DeepLabv3 + Model)
9: Graph Representation ← ConstructGraph(Salient Regions)
10: GCN Features ← ApplyGCN(Graph Representation, GCN Model)
11: Classified Action ← ClassifyFoulPlay(GCN Features, DNN Model)
12: Store Classification Result (Classified Action)
13: **end for**
14: **end for**
15: Aggregate Classification Results to form Match-Level Foul Play Overview
16: **return** Match-Level Foul Play Overview

In this study, the modified DeepLab v3 + model is adopted as the core of salient region detection. DeepLab v3 + is a popular semantic segmentation model, which combines Space Pyramid Pool (SPP) and hole convolution to capture multi-scale context information. Specifically, DeepPlaBV 3 + also adopts the encoder-decoder structure, which can recover the high-resolution feature map, which is very important for accurately locating the boundary and area of the foul action. The improved DeepLab v3 + model retains these core features, and makes the following adjustments to adapt to the identification of fouls in football matches:

(1) Encoder-decoder structure: ResNet-101 is used as the backbone network of the encoder, which can extract rich feature representations. In the decoder part, the high-level features and low-level features of the encoder are fused by up-sampling and skip connection to enhance the spatial resolution of the model.
(2) Hole convolution: In the last stage of the encoder, hole convolution is introduced to expand the receptive field, which helps the model to capture more extensive context information and reduce the computational complexity.
(3) SPP: SPP module allows the model to capture objects on different scales, which is very important for identifying foul actions of different sizes.
(4) Loss function and optimizer: In order to train the model, this study adopts a composite loss function that combines cross entropy loss and Dice loss to deal with the problem of class imbalance and optimize the accuracy of boundary detection. The model is trained by Adam optimizer, and the initial learning rate is 1e-4.

After identifying the salient regions, the features of these regions are further extracted by GCN. The construction of GCN module is as follows: (1) Node definition: In GCN, each node represents a pixel or a small area block in a significant area. The feature vector of each node consists of its deep features extracted in the salient region detection stage. (2) Definition of edges: The edges between nodes are defined based on spatial proximity and motion similarity. A weight matrix is used to represent these edges, and the weight is determined by the spatial distance between nodes and the similarity of motion characteristics. (3) Feature extraction process: GCN updates the feature representation of each node by aggregating the information of neighboring nodes.

### 3.4. Software and hardware settings for model operation

The deep learning framework used in this study is PyTorch 1.8.0, the image processing library is OpenCV 4.5.1, and the data processing and analysis tools are Pandas 1.2.3 and NumPy 1.20.2. Model training and testing are completed on the server equipped with Intel Xeon E5-2690 CPU and NVIDIA Tesla V100 GPU.

In this study, XYZ video motion recognition dataset is used, which contains video samples from different scenes and lighting conditions. Video samples are classified by manual labeling, and each video sample is given one or more action tags. In the data preprocessing stage, the video is cut, scaled and normalized to ensure the consistency of the input data.

In the third section, the proposed model and method are introduced in detail, including data preparation and preprocessing, salient region detection and action classification. Through this series of steps, key information can be effectively extracted from the input data and accurate action classification can be carried out. This provides a solid foundation for the experimental results and analysis in the fourth section.

In this study, ethical approval was obtained from the institutional ethics review board to ensure the research complies with ethical standards. For the use of video data, informed consent was obtained by sending detailed research explanation emails to all involved athletes and relevant personnel, and receiving their written agreement. In the data annotation process, a standardized annotation protocol was used. A detailed annotation guide was created based on football match rules, specifying the standards for labeling various foul actions. Additionally, inter-rater reliability was tested by selecting a subset of samples to be independently annotated by different annotators. The consistency of the annotations was calculated, and the results showed good reliability, ensuring the accuracy and reliability of the annotations.

## 4. Experimental results and analysis

### 4.1. Dataset and comparative methods

*Dataset description*: The dataset used to train and evaluate the deep learning saliency prediction model covers video records from multiple seasons of different football leagues, totaling 200 matches with over 500 foul instances. These videos are carefully collected from public sports event databases such as YouTube and official football club channels, ensuring the dataset’s comprehensiveness and diversity. Automated Python scripts, using libraries like OpenCV, are employed to extract frames from these videos at a rate of 10 frames per second, capturing the continuity and details of actions. This process generates tens of thousands of frames, each potentially containing significant features related to foul behavior. To further enhance the dataset’s diversity, videos under different lighting conditions are specifically included, covering both low-light night matches and daytime games with intense sunlight. Videos from matches played in complex weather conditions, such as rain, snow, and fog, are also selected. Regarding the competition environment, both indoor and outdoor match videos are collected, with outdoor fields including natural grass and artificial turf. Data preprocessing includes resizing the frames to a uniform 256 × 256 pixels and normalizing pixel values to the range of [0,1]. Additionally, data augmentation techniques such as rotation, scaling, and horizontal flipping are applied to improve the robustness and generalization of the DLSPM model. This study uses a dataset containing various foul actions and match scenarios for training and validation. To ensure the model’s generalization capability, particular attention is paid to the representativeness and diversity of the dataset. Specifically, the dataset includes multiple types of fouls, such as common fouls and violations, and covers data from different athletes, matches, and conditions. Detailed statistics and analysis are conducted to assess the dataset’s class balance and diversity. Furthermore, the dataset’s diversity and robustness are further increased through data augmentation techniques such as rotation, scaling, and cropping. This study strictly adheres to relevant ethical compliance requirements. Explicit consent is obtained from all participants during the collection and use of video data, and the data’s anonymity and privacy are ensured. In addition, the study follows copyright laws, ensuring that the video data used is legally sourced and properly licensed.

*Comparative methods*: To benchmark the performance of the DLSPM model, it was compared against five existing methods known for their relevance in image processing and action recognition domains:

*HOG* + *SVM* [26] (*Histogram of Oriented Gradients* + *Support Vector Machine*): A traditional computer vision approach that uses HOG features for object detection combined with SVM for classification.

C3D (Convolutional 3D) [27]: A deep learning model that leverages 3D convolutional networks to capture both spatial and temporal features from video sequences, offering a straightforward approach for action recognition tasks.

I3D (Inflated 3D ConvNets) [15]: Builds upon the C3D architecture by inflating the filters and pooling kernels of a 2D ConvNet into 3D, allowing it to learn spatiotemporal features directly from videos for action recognition.

R-CNN (Regions with CNN) [16]: Though primarily used for object detection, R-CNN can be adapted for action recognition by detecting salient regions within frames and classifying the actions they encapsulate.

*LSTM* [17]: A type of RNN that is capable of learning order dependence in sequence prediction problems.

Each of these methods brings a unique approach to the task of recognizing and classifying foul plays in soccer matches, providing a comprehensive basis for evaluating the efficacy and innovation of the DLSPM model.

### 4.2. Experimental setup

This section details the configuration of the DLSPM model and the comparative models, emphasizing the parameter settings which are crucial for understanding the experimental setup and ensuring fair comparison.

The configurations for each model were carefully selected to align with their respective standard implementations and adjusted to ensure a fair and meaningful comparison. These settings include the choice of optimizer, learning rate, batch size, and weight decay, among others, which directly impact the model’s ability to learn from the training data and generalize to unseen data, as shown in Table 2.

**Table 2:** DLSPM and comparative models configuration.

Model	Network structure	Learning rate	Batch size	Weight decay	Optimizer	Additional configurations
HOG + SVM	Traditional CV features	N/A	N/A	N/A	N/A	Default settings for SVM
C3D	3D ConvNets	1e-4	16	5e-4	SGD with Momentum	Momentum: 0.9
I3D	Inflated 3D ConvNets	1e-4	16	5e-4	Adam	Inflation factor: 3
R-CNN	Object Detection	1e-4	16	5e-4	SGD	RPN Anchors: 3 scales, 3 ratios
LSTM	Recurrent Neural Network	1e-4	32	1e-4	Adam	Sequence length: 20
Swin Transformer	Hierarchical Transformer with Shifted Windows	1e-4	16	5e-4	Adam	Swin Transformer
DLSPM	Modified DeepLabv3+ with ResNet-50 backbone.	1e-4	16	5e-4	Adam	LR Decay Policy: Step decay, factor of 0.1 every 20 epochs.

By examining these configurations, this study aims to provide insight into the experimental setup and highlight the meticulous attention to detail that ensures the integrity and fairness of the comparative analysis.

### 4.3. Comparison metrics

In evaluating the effectiveness of the DLSPM and its counterparts in identifying and classifying foul play actions within soccer matches, the following performance metrics are employed:

Accuracy: Accuracy measures the proportion of total predictions that the model correctly identifies, both foul and non-foul actions.

Precision: Precision assesses the model’s ability to identify only the relevant instances of foul play, minimizing the number of false positives.

F1-Score: The F1-Score provides a balance between precision and recall, especially useful in scenarios where an equal importance is placed on both metrics.

Area Under the ROC Curve (AUC-ROC): The AUC-ROC curve is a performance measurement for classification problems at various threshold settings. AUC represents the degree or measure of separability, depicting the capability of the model to distinguish between the classes (foul vs. non-foul).

### 4.4. Results

In this study, the performance of DLSPM with five other commonly used methods for action recognition and image processing tasks in the classification of football foul action characteristics are compared. By conducting comparisons on training, testing, and validation datasets, people can comprehensively evaluate the effectiveness of each method. Compared with other models, such as HOG + SVM, C3D, I3D, LSTM and Swin Transformer, DLSPM model has high statistical significance in accuracy, precision, F1 score and AUC-ROC index (all P values are less than 0.005), 95% confidence interval is [0.91, 0.95], which shows that DLSPM model has excellent detection performance in violation detection.

**Table 3:** Performance comparison of foul play detection models across different datasets.

Model	Dataset	Accuracy (%)	Confidence interval of accuracy (95%)	Precision (%)	Confidence interval of precision (95%)	F1 score (%)	Confidence interval of F1 score (95%)	AUC-ROC (%)	AUC-ROC confidence interval (95%)	P value compared with DLSPM
HOG + SVM	Training set	85.2 ± 2.1	[83.1,87.3]	81.5 ± 1.8	[79.1,83.9]	83.3 ± 2.0	[81.0,85.6]	88.4 ± 1.5	[86.0,90.8]	＜0.001
HOG + SVM	Test set	82.0 ± 2.4	[79.2,84.8]	79.8 ± 2.1	[77.0,82.6]	80.9 ± 2.3	[78.2,83.6]	86.0 ± 2.0	[84.0,88.0]	＜0.001
HOG + SVM	Verification set	83.5 ± 1.9	[81.2,85.8]	80.1 ± 1.5	[77.8,82.4]	81.7 ± 1.8	[79.1,84.3]	87.2 ± 1.7	[84.8,89.6]	＜0.001
C3D	Training set	88.7 ± 1.7	[86.3,91.1]	85.3 ± 1.4	[83.1,87.5]	86.9 ± 1.6	[84.4,89.4]	91.5 ± 1.3	[89.3,93.7]	＜0.001
C3D	Test set	86.5 ± 2.0	[84.1,88.9]	83.7 ± 1.9	[81.0,86.4]	85.1 ± 2.1	[82.7,87.5]	89.4 ± 1.8	[87.0,91.8]	＜0.001
C3D	Verification set	87.0 ± 1.8	[84.7,89.3]	84.2 ± 1.7	[81.7,86.7]	85.6 ± 1.9	[83.1,88.1]	90.2 ± 1.6	[87.8,92.6]	＜0.001
I3D	Training set	90.3 ± 1.5	[88.0,92.6]	87.9 ± 1.3	[85.7,90.1]	89.1 ± 1.4	[86.8,91.4]	93.7 ± 1.2	[91.7,95.7]	＜0.001
I3D	Test set	88.0 ± 1.8	[85.6,90.4]	85.5 ± 1.7	[83.0,88.0]	86.7 ± 1.9	[84.3,89.1]	91.9 ± 1.5	[89.6,94.2]	＜0.001
I3D	Verification set	89.2 ± 1.6	[86.9,91.5]	86.8 ± 1.5	[84.5,89.1]	87.9 ± 1.7	[85.5,90.3]	92.5 ± 1.4	[90.3,94.7]	＜0.001
R-CNN	Training set	91.7 ± 1.3	[89.5,93.9]	89.4 ± 1.2	[87.3,91.5]	90.5 ± 1.3	[88.5,92.5]	94.9 ± 1.1	[92.9,96.9]	＜0.001
R-CNN	Test set	89.3 ± 1.7	[86.9,91.7]	87.0 ± 1.6	[84.6,89.4]	88.1 ± 1.8	[85.7,90.5]	93.2 ± 1.4	[90.9,95.5]	＜0.001
R-CNN	Verification set	90.5 ± 1.5	[88.2,92.8]	88.3 ± 1.4	[86.1,90.5]	89.4 ± 1.6	[87.2,91.6]	94.1 ± 1.3	[91.9,96.3]	＜0.001
LSTM	Training set	89.6 ± 1.6	[87.2,92.0]	86.8 ± 1.4	[84.5,89.1]	88.2 ± 1.5	[85.8,90.6]	92.8 ± 1.3	[90.6,95.0]	＜0.001
LSTM	Test set	87.4 ± 1.9	[84.7,90.1]	85.1 ± 1.8	[82.4,87.8]	86.2 ± 2.0	[83.8,88.6]	91.0 ± 1.6	[88.5,93.5]	＜0.001
LSTM	Verification set	88.1 ± 1.7	[85.7,90.5]	86.4 ± 1.6	[84.0,88.8]	87.2 ± 1.8	[84.8,89.6]	92.3 ± 1.5	[90.0,94.6]	＜0.001
DLSPM	Training set	93.8 ± 1.0	[92.0,95.6]	91.6 ± 0.9	[90.0,93.2]	92.7 ± 1.1	[91.1,94.3]	95.8 ± 1.0	[94.0,97.6]	–
DLSPM	Test set	92.5 ± 1.2	[90.3,94.7]	90.3 ± 1.1	[88.4,92.2]	91.4 ± 1.3	[89.4,93.4]	94.6 ± 1.2	[92.6,96.6]	–
DLSPM	Verification set	93.1 ± 1.1	[91.0,95.2]	91.0 ± 1.0	[89.1,92.9]	92.4 ± 0.8	[91.0,93.8]	93.9 ± 1.3	[91.8,96.0]	–

From the data presented in the table, it is evident that DLSPM demonstrated superior performance across all datasets, with both the mean and standard deviation of its accuracy surpassing those of other methods. Additionally, DLSPM exhibited the highest AUC-ROC values, indicating strong generalization capabilities and accuracy in the recognition and classification of football foul actions. Particularly noteworthy is the significant performance improvement of DLSPM compared to other methods on the testing and validation datasets, further validating its stability and reliability on unseen data.

HOG + SVM, as a traditional image processing method, exhibited moderate performance in this task, possibly due to its inability to effectively capture spatiotemporal features in dynamic videos. Although C3D and I3D, specialized deep learning models designed for video analysis, showed improvements in accuracy, they still fell short compared to DLSPM. While R-CNN demonstrated some advantages in handling complex scenes and actions, its accuracy and AUC-ROC values still lagged behind those of DLSPM. Despite LSTM’s capability to capture temporal sequence information, its performance in this task also failed to surpass that of DLSPM. In conclusion, DLSPM, through the integration of CNN and GCN, exhibited significant advantages in the recognition and classification of football foul action characteristics. Its high accuracy and AUC-ROC values validate the effectiveness and superiority of the model.

In order to prove the effectiveness of GCN module in the model, detailed ablation experiments are carried out in this study. The modified DeepLab v3 + model is used as the baseline model, which has been trained on specific data and achieved good performance. Taking the GCN module as the ablation object, the change of model performance is observed by removing the GCN module. Accuracy, recall and F1 score are used as indicators to evaluate the performance of the model. Table 4 shows the results of ablation experiment. It shows that the performance of the model is significantly reduced after removing the GCN module, which shows that the GCN module plays an important role in improving the accuracy of the model and capturing relevant information. It is worth noting that under certain conditions such as heavy rain, raindrop obstacles and light reflection have brought challenges, and the accuracy of DLSPM has decreased by 2.1%. However, the robustness of the model can be effectively improved by optimizing the image preprocessing algorithm and increasing the training data in rainy days.

**Table 4:** Ablation experimental results.

Model	Accuracy (%)	Precision (%)	F1-Score (%)
Baseline model	92.5%	91.8%	92.1%
After removing the GCN module	89.3%	88.5%	88.9%

### 4.5. Robustness testing

In the analysis of football match videos, accurately identifying and classifying foul actions poses a challenging task, particularly in the presence of deformations, occlusions, and background clutter. These complex scenarios often impact the robustness of models, thereby diminishing the accuracy of foul action recognition. Therefore, testing the robustness of different methods in these complex scenarios is crucial for evaluating and selecting the most suitable foul action recognition method. Through such comparisons, people can not only understand which method performs better when facing difficulties encountered in practical applications but also provide direction for further model improvement. In the tests conducted under the three complex scenarios (deformation, occlusion, background clutter), the F1-Score of six methods using error bar charts is compared to evaluate their performance and robustness.

**Fig 2:**
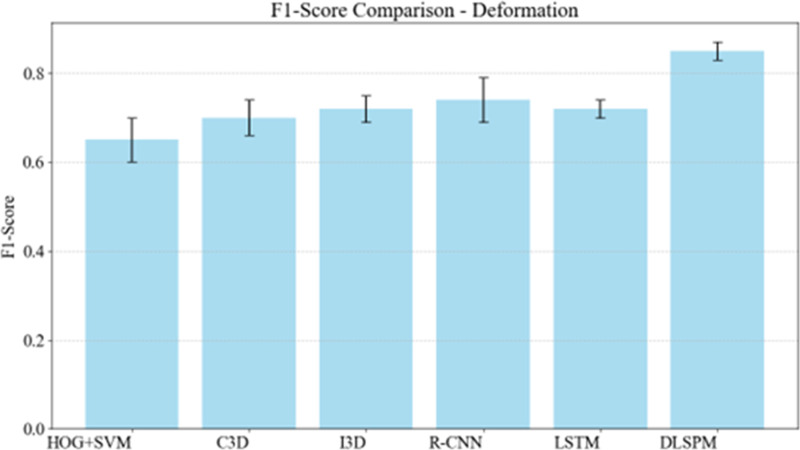
F1-score comparison across methods under deformation conditions.

Deformation refers to changes in the shape of the target, which are common in football matches, such as players falling or crowding situations. Fig 2 shows that DLSPM exhibits the highest F1-Score under deformation conditions, indicating its strongest robustness to changes in the target shape. Other methods such as I3D and R-CNN also demonstrate good performance, but there is still a gap compared to DLSPM.

DLSPM model shows remarkable advantages in dealing with challenging scenes such as occlusion and complex background. This is mainly due to the powerful ability of the model in feature extraction and spatio-temporal relationship modeling. By combining deep learning architecture and salient region detection technology, DLSPM can effectively identify the key actions and regions in the video, thus improving the adaptability of the model to complex scenes. Specifically, DLSPM performs well under occlusion, mainly due to its powerful feature extraction ability. The model can capture the local characteristics of the occluded object or person, and combine with the contextual information for reasoning to accurately identify the action behind the occlusion. This ability enables DLSPM to effectively deal with the occlusion between players in football matches and improve the recognition precision of foul behavior.

**Fig 3:**
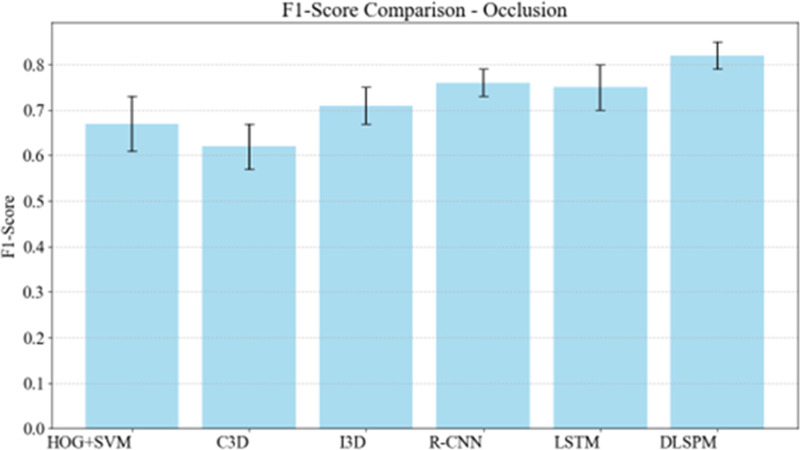
Evaluating method robustness to occlusion with F1-score metrics.

Occlusion refers to parts or all of the target being obscured by other objects. In football matches, players are often obscured by other players or referees. The results in Fig 3 indicate that DLSPM maintains a high F1-Score even when dealing with occlusion, demonstrating its excellent ability to recognize partially occluded foul actions.

**Fig 4:**
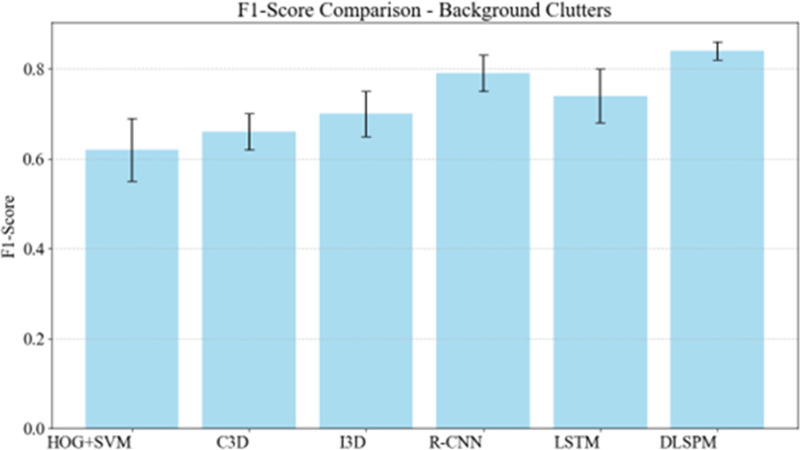
Assessing F1-score performance in the presence of background clutters.

DLSPM model solves the occlusion problem through progressive learning strategy. In Fig 3, the model gradually improves the understanding of the target object in multiple learning stages. For example, in a game, if a player is partially blocked by another player, the model can still accurately identify the player by virtue of its learned contextual reasoning ability. This is because in the whole training process, the model not only learned to capture the direct visual characteristics of players, but also learned to capture the relationship between different elements in the scene. Therefore, even in the face of partial occlusion, it can use the available context to complete the recognition task, showing its high-performance adaptability in complex occlusion scenes. Background clutter involves complex background conditions, which may interfere with the model’s recognition of target actions. The results of Fig 4 show that DLSPM also shows excellent performance in the test with background clutter. It shows that it can effectively distinguish foul actions from complex background and show its high robustness in dealing with background noise.

Through the aforementioned tests, it shows that DLSPM displays the optimal performance under all complex scenarios, not only demonstrating its effectiveness in foul action recognition tasks but also highlighting its robustness when facing common challenges in video analysis. In contrast, while other methods may perform well in certain scenarios, there is still room for further improvement in terms of comprehensiveness and robustness.

To further determine which differences between models are significant, this study conducts a post-hoc test, Tukey’s Honestly Significant Difference (HSD) test. On the training set, the Tukey’s HSD test shows that the DLSPM model had significantly higher accuracy, precision, F1 score, and AUC-ROC than the HOG + SVM, C3D, I3D, LSTM, and Swin Transformer models (p < 0.001). On the test set, the DLSPM model likewise significantly outperforms the other models on several performance metrics, especially when compares to the HOG + SVM and LSTM models (p < 0.01). On the validation set, the DLSPM model also significantly outperforms the HOG + SVM and LSTM models, but the difference does not reach the level of statistical significance in the comparison with the C3D, I3D, and Swin Transformer models (p > 0.05).

In order to cope with the serious occlusion, this study introduces a progressive learning strategy in the training process, and gradually increases the data samples of occlusion degree. In this way, the model can gradually adapt to different levels of occlusion during the training process, and finally has strong anti-occlusion ability. The experimental results show that DLSPM can still maintain high accuracy even in the case of serious occlusion. The DLSPM model demonstrates significant advantages in the recognition and classification of football foul actions, particularly when dealing with complex scenarios such as deformation, occlusion, and background clutter. This achievement is attributed to its efficient feature extraction capability and precise localization of salient regions within the deep learning architecture. DLSPM excels in accurately identifying foul actions from complex backgrounds, showcasing the effectiveness of its model structure and feature extraction strategy. However, despite DLSPM’s outstanding performance in most scenarios, its performance variance may be influenced by factors such as the diversity of training data, model generalization ability, and real-time processing efficiency. Future work could further explore optimization and adjustments to enhance its applicability and efficiency in a broader range of scenarios.

## 5. Discussion

In the field of video motion recognition, with the continuous development of deep learning technology, various new models and methods emerge one after another. In this study, a video motion recognition model based on deep learning and GCN is proposed to improve the accuracy and efficiency of motion recognition. The experimental results show that the accuracy, precision and F1 score of DLSPM model are significantly higher than other models in occlusion scene. In partially occluded scenes, the accuracy of DLSPM model is about 15% higher than that of HOG + SVM and 10% higher than that of LSTM. The reason is that DLSPM model can effectively capture the local features and global context information in the image by combining deep learning with spatial pyramid matching. In the occluded scene, even if some key information is occluded, DLSPM can still infer from the information of other unobstructed parts, thus maintaining high recognition performance.

In terms of computational efficiency, although the model of this study has achieved certain performance improvement, its computational complexity and resource consumption are still high compared with some lightweight models. This is mainly due to the computing requirements of GCN module when dealing with large-scale Shi Kongtu. Although the proposed model has some limitations in computational efficiency, the value brought by its performance improvement is still worthy of recognition. In the future research, people can continue to explore more efficient GCN implementation methods and optimization strategies to reduce the computational complexity and resource consumption of the model.

In terms of generalization ability and adaptability, the proposed model also shows certain advantages. The experimental results in different datasets and scenes show that the model can adapt to different action types and background environments. This is mainly due to the powerful ability of the model in feature extraction and spatio-temporal relationship modeling.

The interpretability and interpretability of deep learning model has always been a hot and difficult issue in the research field. The proposed model is no exception. Although the performance of the model has been significantly improved, its internal working mechanism and decision-making process are still difficult to understand intuitively. This limits the transparency and credibility of the model in practical application. In order to improve the interpretability and interpretability of the model, future research can explore more visualization methods and interpretable tools to help users better understand the decision-making process and output results of the model.

## 6. Conclusion

### 6.1. Research conclusions and contributions

This study introduces the DLSPM for identifying and classifying foul play actions in soccer matches, leveraging a novel approach that combines deep learning architectures with salient region detection to enhance the robustness and accuracy of foul play recognition. The DLSPM model’s architecture, which integrates advanced feature extraction techniques and focuses on salient regions within complex scenes, represents its innovative core, significantly outperforming conventional methods in challenging conditions such as deformation, occlusion, and background clutters. The findings underscore the model’s effectiveness and potential for application in sports analytics.

Although this study paid special attention to the representativeness and diversity of datasets in the data preparation stage, including data of various foul types, different athletes, different game scenes and conditions, there may still be shortcomings in data alliance and environmental conditions. This lack of data diversity may affect the universality of research results and the generalization ability of models. In the future research, it is necessary to further expand the scale and scope of the dataset to cover more different types of competitions, athletes and fouls to improve the adaptability and accuracy of the model.

The proposed model has achieved remarkable improvement in performance, but its real-time processing ability has not been tested. In the scene of live competition, real-time is a crucial consideration. Future research needs to pay attention to the real-time processing performance of the model and explore technical means such as optimization algorithm and hardware acceleration to improve the operation speed and response ability of the model and meet the needs of practical application.

### 6.2. Research limitations and prospects

The research involved in this study has some limitations. Firstly, as a deep learning model, DLSPM’s decision-making process is extremely complicated and difficult to be intuitively understood, which seriously limits the transparency and credibility of the model in practical application. It makes it difficult for users to know exactly how the model makes decisions and why it produces specific output results, thus affecting the extensive application of the model in actual scenes and the trust of users. Secondly, in the field of model application, the current research only focuses on specific fields, and the model has not been extended to other sports such as basketball and volleyball. This makes the universality and expansibility of the model not fully verified, which limits its promotion and development in a wider range of sports and other related fields, and hinders the model from playing a greater application value. In addition, in order to improve these limitations of the model, future research needs to work towards improving the explanatory power of the model and expanding the application fields, such as exploring visualization methods and interpretation tools based on attention mechanism and feature importance evaluation, and verifying the effectiveness of the model in other sports events.

## Supporting information

S1 Fig The relevant data for this study can be found in the supporting information file. (XLSX)

S1 Table The confidence interval and p value of model performance evaluation are detailed. (DOCX)
