# Supplementary material for: A method for feature division of Soccer Foul actions based on salience image semantics
Source: PLoS One. 2025 Jun 13;20(6):e0322889. doi: 10.1371/journal.pone.0322889 (PMC12165423; doi:10.1371/journal.pone.0322889)
Supplement: S1 Table — (DOCX) [file pone.0322889.s002.docx]

**Supplementary Table S1**

Table S1: Confidence interval and p value of model performance evaluation

| Experiment/analysis | Confidence interval (95%) | P value (compared with DLSPM) |
| --- | --- | --- |
| HOG+SVM | Accuracy: [79.2, 84.8] % | p < 0.001 |
|  | F1-score: [78.2, 83.6] % | p < 0.001 |
| C3D | Accuracy: [84.1, 88.9] % | p < 0.001 |
|  | F1-score: [82.7, 87.5] % | p < 0.001 |
| I3D | Accuracy: [85.6, 90.4] % | p < 0.001 |
|  | F1-score: [84.3, 89.1] % | p < 0.001 |
| R-CNN | Accuracy: [86.9, 91.7] % | p < 0.001 |
|  | F1-score: [85.7, 90.5] % | p < 0.001 |
| LSTM | Accuracy: [84.7, 90.1] % | p < 0.001 |
|  | F1-score: [83.8, 88.6] % | p < 0.001 |
